# Noise Reduction of Welding Crack AE Signal Based on EMD and Wavelet Packet

**DOI:** 10.3390/s20030761

**Published:** 2020-01-30

**Authors:** Kuanfang He, Zixiong Xia, Yin Si, Qinghua Lu, Yanfeng Peng

**Affiliations:** 1 School of Mechatronics Engineering, Foshan University, Foshan 528000, China; xiazx96@163.com (Z.X.); sy987110@163.com (Y.S.); qhlu@fosu.edu.cn (Q.L.); 2 Hunan Provincial Key Laboratory of Health Maintenance for Mechanical Equipment, Hunan University of Science and Technology, Xiangtan 411201, China; pyf1988@sina.com

**Keywords:** welding crack, AE signal, noise reduction, EMD, wavelet packet

## Abstract

The acoustic emission (AE) signal collected by a sensor in the welding process has an overlapping frequency band and weak characteristics under a complex noise background. It is difficult for the wavelet noise reduction method, with single basis function, to effectively match the different characteristic information of the welding crack AE signal. Taking into account the adaptive decomposition characteristics of Empirical Mode Decomposition (EMD), a novel wavelet packet noise reduction method for welding AE signal was proposed. The welding AE signal was adaptively decomposed into several Intrinsic Mode Functions (IMFs) by the EMD. The effective IMFs were selected by the frequency distribution characteristics of the welding crack AE signal. A wavelet packet, with a specific basis function, was subsequently performed on the effective IMFs, which were reconstructed to be the welding crack AE signal. The simulated and experimental results indicated that the proposed method can effectively achieve noise reduction of the welding crack AE signal, which provided a mean for structure crack detection in the welding process.

## 1. Introduction

The acoustic emission (AE) signal is a phenomenon of transient elastic waves caused by a change in external conditions (stress, temperature, etc.) on a part of a structure [1]. By way of sensor detection, the method of recording and analyzing AE signals to determine the degree of internal damage in a structure is called AE detection technology [2]. The AE signal is an elastic wave released by energy within a material, which can characterize the physical phenomena essence of welding crack generation and extension [3,4,5]. Since there are many other AE signal sources, such as friction and welding arc impact, the actual collected welding crack AE signal has an overlapping frequency band and weak characteristics under a complex noise background [6]. In order to achieve the online detection of welding structure cracks, it is necessary to obtain the AE signal of a crack source under a complex noise background. Therefore, the noise reduction of AE signals during the welding process is the key to achieving the AE detection of welding structure cracks.

Traditional noise reduction methods include Fast Fourier Transformation(FFT) [7], wavelet analysis [8], wavelet packet analysis [9], empirical mode decomposition (EMD) [10], etc. Since an AE signal is a nonlinear and non-stationary signal, the FFT method, based on a linear and stationary assumption, cannot fully obtain the necessary information [11]. Wavelet analysis can achieve noise reduction of non-stationary signals with multi-resolution characteristics; however, wavelet decomposition is aimed at a low frequency signal, which cannot involve the high frequency part. It is difficult for the wavelet analysis to satisfy both the high and low frequency resolutions [12,13]. Based on the good characteristics of wavelet orthogonal basis, wavelet packets improve the resolution of the high frequency part, which has a strong local analysis ability for the non-stationary signal. The EMD technique is a key technology for the Hilbert Huang transform (HHT), which decomposes the signal adaptively into a series of Intrinsic Mode Functions (IMFs) containing different scales. The EMD is suitable for analyzing nonlinear and non-stationary signals [14]. However, the existing methods mainly adopt EMD to decompose the vibration signal, and then perform wavelet packet transforms to the decomposed components by the single wavelet basis. The wavelet packets with the appropriate basis function was not considered to process AE signals according to the characteristics of each decomposed component. The actual collected welding crack AE signal accompanies other parts, which have the overlapping frequency band and weak characteristics. The noise reduction by wavelet packet decomposition with single basis function is difficult to match to the different characteristic information of the welding crack AE signal with other parts. EMD is capable of modal aliasing at the high frequency section, which has difficulty in effectively achieving a noise reduction of welding crack AE signals under a complex noise background. Thus, it is difficult to effectively extract the welding crack AE signals from the complex noise background by traditional signal noise reduction methods, such as wavelet packets or EMD. 

Taking into account the adaptive decomposition characteristics of the EMD, a signal can be decomposed into several components. Thus, a wavelet packet with multiwavelet functions can be performed on the different components, which can meet the requirements of symmetry, orthogonality, compactness and higher order vanishing moments simultaneously. Therefore, combining the advantages of EMD and a wavelet packet, the actual collected welding crack AE signals are decomposed into a series of IMFs adaptively, then each IMF is further processed by a wavelet packet with specific basis function. The benefit of this processing is to realize the multiple wavelet basis functions for matching different characteristic information of welding crack AE signals. Therefore, a novel method based on EMD and a wavelet packet is proposed to achieve the noise reduction of the welding crack of the AE signal. The aim of the noise reduction method based on EMD and wavelet packets is to avoid the defect that a single wavelet basis has in effectively matching the different characteristics of the signals. The other aim is to improve EMD decomposition capability, which provides a new extraction method for AE signals under complex noise background. Based on the AE simulation signal, the denoising effect of the proposed method is compared to EMD and wavelet packets respectively. Meanwhile, the proposed method is also applied to the welding crack AE signal for verification of validity. The organization of paper is as follows: In Section 2, the algorithm of EMD is introduced, in Section 3 the theory and algorithm of the noise reduction by wavelet packets are introduced. In Section 4, the principles and methods of the noise reduction method based on EMD and wavelet packets are discussed for the welding crack AE signal. In Section 5, the proposed method is applied to noise reduction of a welding crack AE signal. The conclusions are summarized in Section 6.

## 2. EMD

The EMD method is a new time-frequency analysis method, which is widely used in mechanical fault diagnosis [15], feature extraction [16], gear crack signal analysis [17] and other fields. The signal is decomposed into a number of IMFs based on the time scale characteristics. The given original signal x(t) is decomposed by EMD [16] as follows:

(1) $$x(t)=\sum_{i=1}^{n}IMFi(t)+rn(t)$$

where IMFi(t) represents the IMF component, rn(t) represents residual component.

Equation (1) shows that the EMD method can decompose a signal into a series of IMF components and a residual component.

## 3. Noise Reduction by Wavelet Packet

### 3.1. Definition of Wavelet Packet

The wavelet packet approximation expression can be obtained by the multi-resolution analysis of the square integrable real space as following.

(2) $$L^{2}(R)=⊕W_{-1}⊕W_{0}⊕W_{1}⊕=\underset{f\in z}{⊕}W_{j},\forall j\in Z$$

where the space of wavelet function is $W_{j}$, the scale factor is j, ⊕ is the orthogonal sum of the two subspaces. Equation (2) means that the space of the real number, $L^{2}(R)$, is the orthogonal sum of wavelet subspace $W_{j}(j\in z)$ according to different scale factors. Wavelet packet analysis can improve the frequency resolution by subdividing the frequency band in binary form.

A time series ${\left\{hn\right\}}_{n\in z}$ is given to be satisfied as follows.

(3) $$\left\{ \begin{array}{l} \sum_{n}hn-2khn-2l=\delta k,l\\ {\sum_{n}hn=2}^{1/2}\\ gk={\left(-1\right)}^{k}hl-k \end{array}\right.$$

where hk and gk are the filter coefficients.

$w_{n}=L^{2}(R),n=1,2$ is a group of recursive functions generated by scaling functions φ(t) and wavelet functions ϕ(t), and the following relations are established.

(4) w0(t)=φ(t),w1(t)=ϕ(t)

(5) $$w2n=2^{1/2}\sum_{k}hkwn(2t-k)$$

(6) $$w2n+1(t)=2^{1/2}\sum_{k}gkwn(2t-k)$$

The geometric function ${\left\{w_{n}(t)\right\}}_{n\in z}$ is defined by Equations (4), (5) and (6), which is determined as the wavelet packet by $w_{0}(t)=\varphi (t)$. Thus, wavelet packet ${\left\{w_{n}(t)\right\}}_{n\in z}$ can be defined as function aggregation with certain relation between $w_{0}(t)$ and $w_{1}(t)$.

### 3.2. Decomposition and Reconstruction of Wavelet Packets

Wavelet packet decomposition divides the frequency band into multiple levels, and further decomposes the high frequency part that is not subdivided by wavelet analysis. Wavelet packet decomposition selects adaptively the corresponding frequency band to match the spectrum characteristic of the signal, which improves the time frequency resolution. The wavelet packet decomposes the signal into the corresponding frequency band components according to the random time frequency resolution.

**Figure 1:**
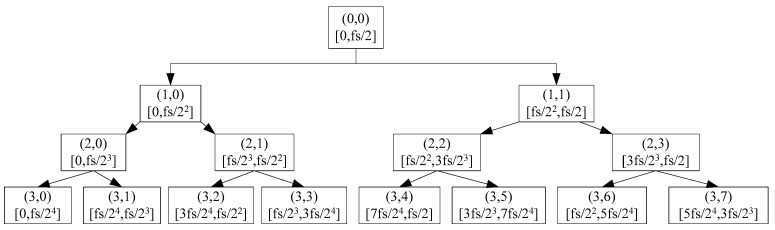
The three-layer wavelet packet decomposition tree and the frequency band division.

The x(t) is assumed as a one-dimensional signal with noise, the three-layer wavelet packet decomposition was taken as an example. The structure of the three-layer wavelet packet decomposition tree and the schematic diagram of the frequency band division are shown in Figure 1.

In Figure 1, the *f*_s_ $f_{s}$ is the sampling frequency, the node (0,0) is the signal to be decomposed, the node (*i*,*j*) is the *j* group coefficient of the *i* layer decomposition. The *S*(*i*,*j*) is assumed as the reconstruction coefficient of the wavelet packet node, the signal has the following relationship after the three-layer decomposition by the wavelet packet.

(7) x(t)=S(3,0)+S(3,1)+S(3,2)+S(3,3)+S(3,4)+S(3,5)+S(3,6)+S(3,7)

### 3.3. Soft Threshold Denoising of Wavelet Packet

In wavelet packet analysis, the noise reduction algorithm of the signal was basically the same as that of the wavelet analysis. The difference is that wavelet packet analysis decomposed the low and high frequency part at the same time, which had more accurate local analysis ability.

Generally, the basic steps of wavelet packet noise reduction were as follows:

(a) The basis function and the layers number were determined to decompose the signal by wavelet packet.
(b) The optimal wavelet packet basis function was determined by using the minimum Shannon entropy criterion.
(c) The threshold quantization was performed to the wavelet packet decomposition coefficient. The threshold was selected according to the minimaxi threshold selection criterion. The decomposition coefficient of the optimal wavelet packet basis was quantified by soft thresholding.
(d) The quantized decomposition coefficient was reconstructed.

Among them, the mathematical expressions of the minimaxi threshold criterion is as follows.

(8) $$\lambda =\left\{ \begin{array}{ll} \sigma (0.3936+0.1829\log_{2}N) & N>32\\ 0 & N<32 \end{array}\right.$$

where λ is the threshold, σ is the mean square of the noise signal, and *N* is the number of the coefficients of the wavelet packet node.

The expression of the soft threshold function is defined as following.

(9) $$\eta (x,\lambda )=\left\{ \begin{array}{ll} 0 & \left|x\right|<\lambda \\ sign(x)(\left|x\right|-\lambda ) & \left|x\right|\geq \lambda \end{array}\right.$$

where *x* is the coefficient of the wavelet packet node.

## 4. Noise Reduction Based on EMD and Wavelet Packet

The EMD originates directly from the signal itself, which has the ability of adaptive decomposition. The main modes of different AE sources can be obtained by EMD performed to the welding AE signals. In the decomposition process, there is a phenomenon of overlapping frequency bands in each mode. There are coexistences of the arc and friction components in the welding crack AE signal. Therefore, the reconstruction of the high frequency IMF components may contain other components. In order to achieve the noise reduction of AE signal in a complex noise background, a noise reduction method based on EMD and wavelet packets was proposed. The main modes of different AE source signals were obtained by EMD. Then the wavelet packet with specific basis function was adopted to match the different characteristic information of each AE source signal. The combination of the EMD and wavelet packets can improve the mode aliasing phenomenon and eliminate noise components. The welding crack AE signal can be extracted effectively under complex noise background.

**Figure 2:**
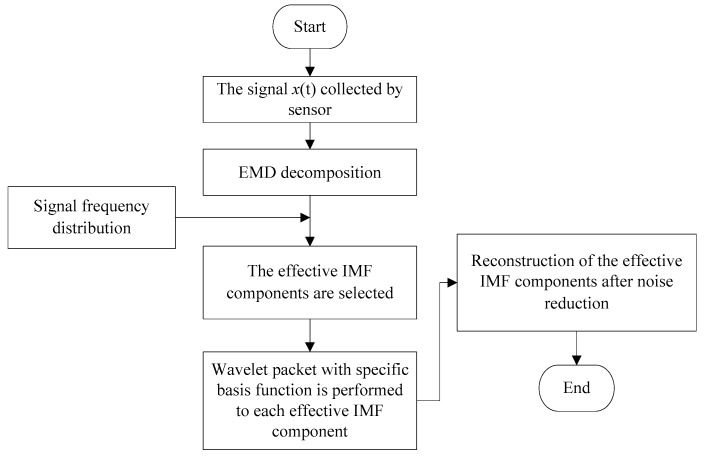
The flow chart of the noise reduction method based on EMD and wavelet packet.

The flow chart of the noise reduction method based on EMD and wavelet packet is shown in Figure 2.

The steps of the algorithm were as follows.

(a) The original AE signal was adaptively decomposed into several IMF components by EMD.
(b) The effective IMF components containing the welding crack mode was selected according to the frequency distribution.
(c) The wavelet packet with different wavelet basis was used to conduct the soft threshold denoising to each effective IMF component.
(d) Reconstruction of the effective IMF components after the noise reduction. The signal after noise reduction was obtained.

In order to verify the validity of the noise reduction method based on EMD and wavelet packets, a simulation AE signal was established. The expression of the simulation signal was as follows.

(10) $$\begin{array}{ll} x(t) & =2[\exp(-2\pi {\left((t-t_{1})/\alpha_{1}\right)}^{2})]\sin(2\pi f_{1}(t-t_{1}))+2[\exp(-2\pi {\left((t-t_{2})/\alpha_{2}\right)}^{2})]\sin(2\pi f_{2}(t-t_{2}))\\ & +2[\exp(-2\pi {\left((t-t_{3})/\alpha_{3}\right)}^{2})]\sin(2\pi f_{3}(t-t_{3})) \end{array}$$

**Figure 3:**
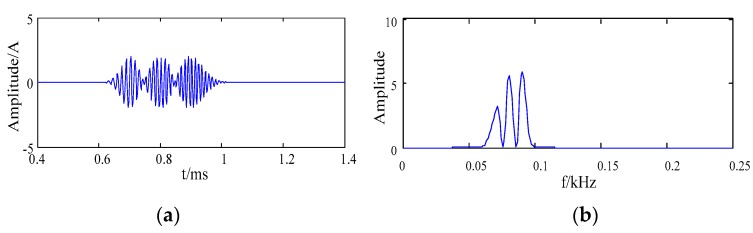
The waveform and the frequency spectrum of the simulation signal (**a**) the waveform; (**b**) the frequency spectrum.

The signal was composed of three pulse signals. Among them, $f_{1}$, $f_{2}$ and $f_{3}$ were the frequencies of the three harmonic signals, the values of parameters were $f_{1}$ = 60 kHz, $f_{2}$= 80 kHz and $f_{3}$ = 100 kHz, $t_{1}$ = 0.7 ms, $t_{2}$ = 0.8 ms, $t_{3}$ = 0.9 ms, $\alpha_{1}$= 0.0001, $\alpha_{2}$ = 0.00015, and $\alpha_{3}$ = 0.0002, the sampling frequency was 500 kHz. The waveform and the frequency spectrum of the simulation signal are shown in Figure 3.

**Figure 4:**
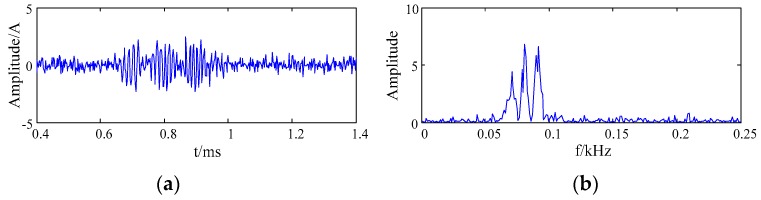
The waveform and the frequency spectrum of the simulation signal with noise (**a**) the waveform; (**b**) the frequency spectrum.

The Gauss white noise of SNR 8 dB was added to the simulation signal, the waveform and the frequency spectrum of the simulation signal are shown in Figure 4.

It can be seen that the AE simulation signal with noise had the appearance of overlapping frequency bands and weak characteristics. It was difficult to identify signal characteristics effectively. Therefore, in order to effectively identify the signal characteristic, it was necessary to conduct noise reduction for the AE simulation signal.

In this paper, the noise reduction by EMD, wavelet packet soft threshold and the proposed methods were applied to the simulation signal with noise. The evaluation standard of the testing result was the Signal to Noise Ratio (*SNR*). The mathematical expression of *SNR* is as follows:

(11) $$SNR=10\mathrm{lg}\left[\frac{\sum_{t=1}^{n}x^{2}(t)}{\sum_{t=1}^{n}{\left(\hat{x}(t)-x(t)\right)}^{2}}\right]$$

where *n* is the length of the AE signal. $\hat{x}(t)$ is the signal after noise reduction. x(t) is the signal without noise.

(1) The noise reduction by EMD

**Figure 5:**
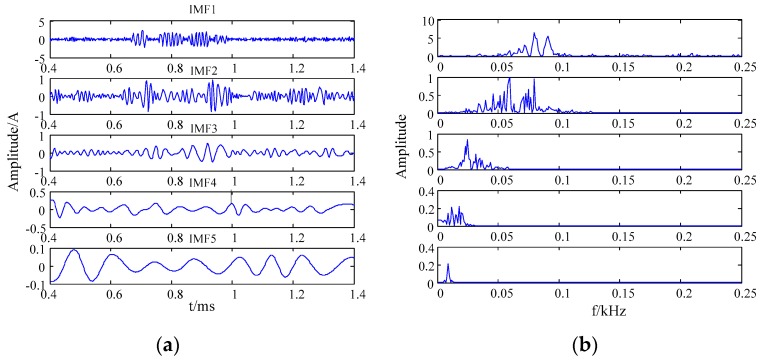
The IMF component and the frequency spectrum of the simulation signal (**a**) the waveform; (**b**) the frequency spectrum.

The finite IMF components were decomposed from the simulation signal by EMD. Among them, the residual components were monotonous and small, which can be ignored. The IMF component waveforms and the frequency spectrum of the simulation signal by EMD are shown in Figure 5.

**Figure 6:**
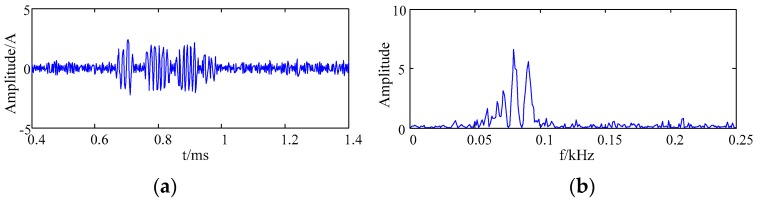
The waveform and spectrum after denoising by EMD (**a**) the waveform; (**b**) the frequency spectrum.

According to the frequency distribution of the simulation signal, it can be seen from Figure 5 that the effective components were IMF1 and IMF2. The effective IMF components were directly combined to be the denoised result of the simulation signal. Figure 6 shows the waveform and frequency spectrum of the denoised simulation signal by EMD.

It can be seen from Figure 4 and Figure 6 that the noise signal was suppressed by the EMD. However, IMF1 and IMF2 components still contained noise. Therefore, the denoising effect of the simulation signal obtained by the EMD method was not remarkable when the IMF1 and IMF2 components were reconstructed directly.

**Figure 7:**
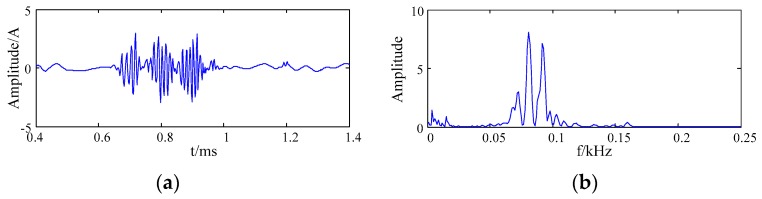
The waveform and spectrum after denoising by wavelet packet (**a**) the waveform; (**b**) the frequency spectrum.

(2) Noise reduction by wavelet packets:

The original signal was decomposed into three layers by a db3 wavelet basis. Figure 7 shows the waveform and frequency spectrum of the simulation signal after denoising by wavelet packet soft threshold method.

It can be seen from Figure 4 and Figure 7 that the noise signal was better suppressed by the wavelet packet soft threshold. Since the single wavelet base function was adopted to the whole signal, it was difficult to match the different feature information in the signal. Therefore, some features of signal were lost after noise reduction by the wavelet packet. The denoised signal was distorted.

**Figure 8:**
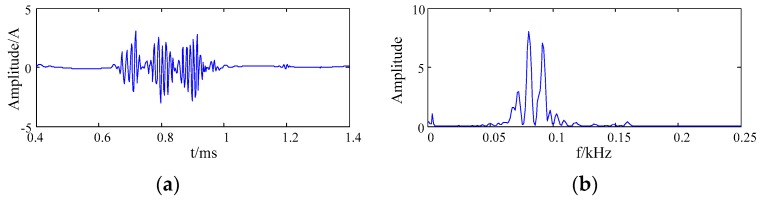
The waveform and spectrum after noise reduction based on EMD and wavelet packet (**a**) the waveform; (**b**) the frequency spectrum.

(3) Noise reduction based on EMD and wavelet packet:

The wavelet packet soft threshold method was used to denoise the IMF1 and IMF2 respectively. The db4 wavelet basis was used to decompose the IMF1 into four layers, and the db3 wavelet basis was used to decompose the IMF2 into three layers. The effective IMF components were denoised by the wavelet packet soft threshold method. The denoised IMF1 and IMF2 components were reconstructed to obtain the destination signal, of which the waveform and spectrum is shown in Figure 8.

**Figure 9:**
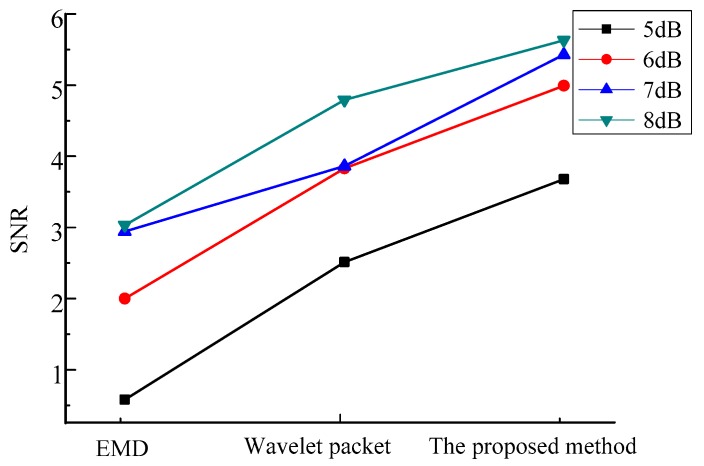
The SNRs of the simulation signal with different levels noise by the three methods.

The Signal to Noise Ratios (SNRs) of the three methods applied to the simulation signal, with different levels of SNRs, are shown in Figure 9.

It can be seen in Figure 9 that the proposed method had better denoising effect with the original signal. The wavelet packets, with multi basis function, could effectively match the different characteristics information of the signal. The decomposition ability of EMD has been improved, as well as the denoising effect.

## 5. Noise Reduction of the Welding Crack AE Signal

**Figure 10:**
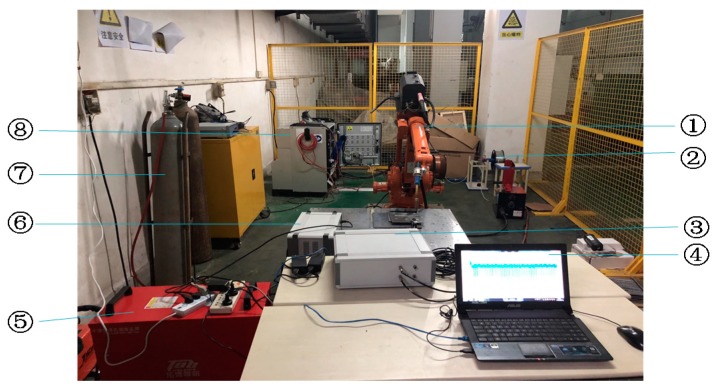
Experiment and testing platform for the double pulse MIG welding, ①Welding robot; ②Welding wire feeding mechanism; ③the are signal acquisition box; ④Computer; ⑤Welding machine; ⑥the arc sensor box; ⑦The argon gas storage tank; ⑧The power supply.

The experimental platform is shown in Figure 10. Experiments were performed by a double pulse Metallic Inert Gas (MIG) welding machine and robot. The material of work piece was a 6061 aluminum alloy with a slab thickness of 5 mm, the argon flow rate is 15 L/min, the elongation of the welding wire was 15 mm, and the diameter of the welding wire was 1.2 mm. The welding parameters are set as shown in Table 1.

**Figure 11:**
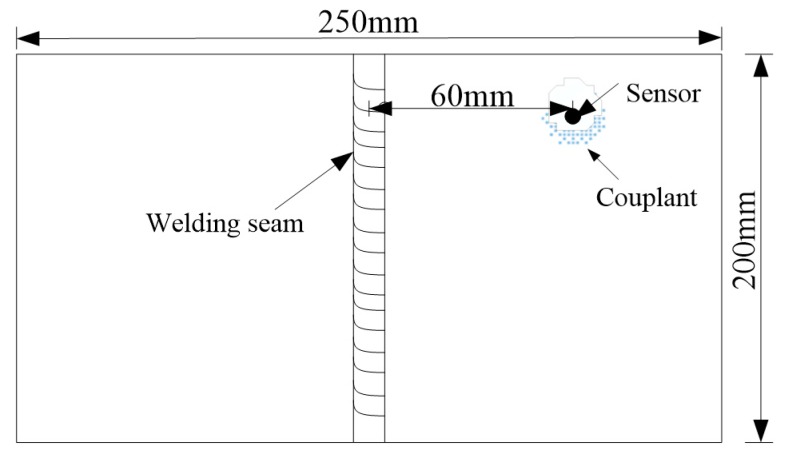
The arranged sensor position.

The DS5-8A AE system (BeiJing Softland Times Scientific&technology Co.LTD, Beijing, China) was used to collect the welding crack AE signal, which provided the experimental data. The DS5-8A AE system was produced by Soft Island Times Technology Co., Ltd. The sensor model was RS-15A, the front amplifier model was Smart AE. The AE sensor installation procedure in the welding process included the following. Firstly, the position of the sensor on the specimen was sanded and smoothed in the process of the welding experiment, and secondly, the sensor was pasted before welding. The distances of the location and the welding seam was 60 mm. Finally, the coupling between the sensor and the specimen was added to avoid a loss of signal caused by the air barrier. The specific sensor placement is shown in Figure 11. In order to effectively collect experimental signals, it was necessary to set up the sampling frequency, hit dimension time, hit locking time, waveform threshold and other relevant parameters of the AE signal system. The related parameter settings are shown in Table 2.

In Table 2, the hit-lock time is the time it takes to stop the collecting operation of the acquisition card. The hit-lock time was set by a positive integer with a range of 1∼+∞ μs. The hit-lock time settings were important. The appropriate parameter selection can play the role of eliminating the reflected wave and other noise. If the parameter selection is too large, the useful signal may be removed as noise. The AE waveform varies with the specimen material, shape, size, and other factors, so the parameters of hit-lock time should be selected according to the actual waveform observed in the sample. The hit-lock time parameter was set to be 2000 μs.

**Figure 12:**
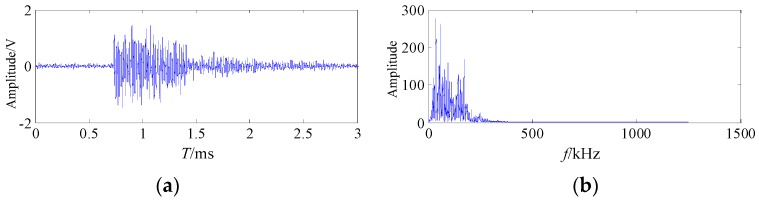
The waveform and spectrum of the welding crack AE signal (**a**) the waveform; (**b**) the frequency spectrum.

The signal waveform related to the crack characteristics was selected to be processed and analyzed. The waveforms and the frequency spectrum of the welding crack AE signal are shown in Figure 12.

During the welding process, the frequency band of the friction AE signal mainly concentrated on 10–63 kHz, the frequency band of the welding arc shock AE signal concentrated on 22.5–103.5 kHz, and the frequency band of the welding crack AE signal mainly concentrated on 100–265.5 kHz [6]. It is shown on Figure 12 that the collected welding crack AE signal mainly contains the friction, welding arc shock and other noises. The collected AE signal in the welding process had overlapping frequency bands, which showed the weak characteristics under the complex noise background.

**Figure 13:**
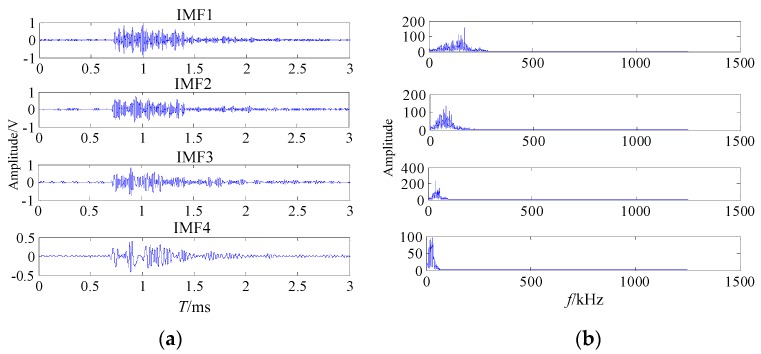
The waveforms and spectra of the first four IMFs (**a**) the waveform; (**b**) the frequency spectrum.

The proposed noise reduction method was performed to the welding crack AE signal. Firstly, the welding crack AE signal was decomposed by EMD. The first four IMF components waveforms and the frequency spectra of the AE signals are shown in Figure 13.

**Figure 14:**
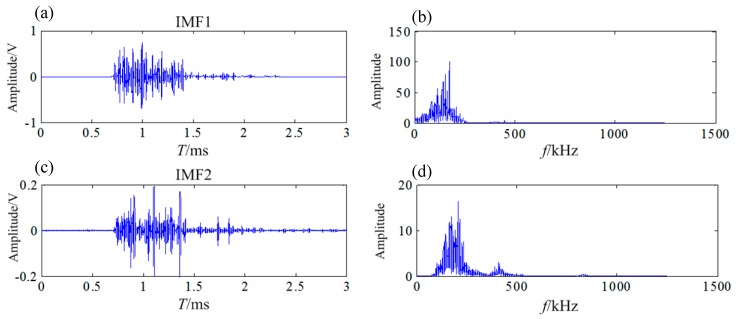
The waveform and spectrum of IMF after denoising (**a**),(**c**) the waveform; (**b**),(**d**) the frequency spectrum.

According to the frequency distribution characteristics of the welding crack AE signal, IMF1 and IMF2 were selected as the effective IMF components of the welding crack AE signal. The db8 wavelet basis was used to decompose the IMF1 into four layers, the db6 wavelet basis was used to decompose the IMF2 into three layers. The effective IMF component waveforms and the spectra are shown in Figure 14.

**Figure 15:**
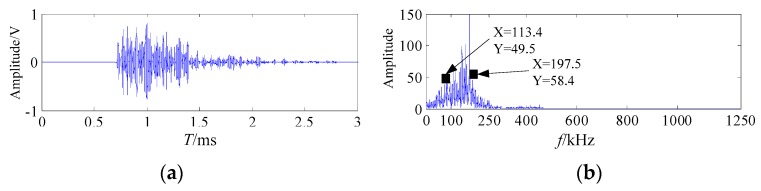
The waveform and spectrum of the welding crack AE signal after noise reduction (**a**) the waveform; (**b**) the frequency spectrum.

The welding crack AE signal is reconstructed by the IMF1 and IMF2 after noise reduction. The waveform and spectrum of the welding crack AE signal are shown in Figure 15.

It can be seen from Figure 12 and Figure 15 that the original welding crack AE signal was denoised by the proposed method. During the welding process, the frequency bands of different excitation source AE signals in literature [6] can be used for the judgment basis for verifying the denoising effect. The frequency band of the friction AE signal mainly concentrated on 10–63 kHz, the frequency band of the welding arc shock AE signal concentrated on 22.5–103.5 kHz, the frequency band of the welding crack AE signal mainly concentrated on 100–265.5 kHz [6]. It is shown in Figure 15 that the frequency band of welding crack AE signal after noise reduction mainly concentrated on 113.4kHz–197.5kHz, which coincided with the results of the literature [6].

## 6. Conclusions

A novel noise reduction method based on EMD and wavelet packets has been proposed. The method adopted the adaptive decomposition characteristics of EMD, which decomposed the welding AE signal into several IMF components. Wavelet packets, with multi basis function, have been subsequently performed to the IMF components respectively. Since the wavelet packets, with multi basis function, matched different feature information of the welding AE signal, the denoising performance had been greatly improved. The denoising performance comparative analysis had been performed on the AE simulation signal. The results showed that the denoising effect of the proposed method was superior to the others.

The proposed method had been also applied to the welding AE signal. The frequency band of the welding crack AE signal after noise reduction mainly concentrated on 111–195 kHz. The result indicated that the proposed method can effectively achieve noise reduction of the welding crack AE signal. Due to the wavelet packets, with multi basis function, effectively matching the different characteristics information of the welding AE signal, the decomposition ability of EMD has been also improved. The proposed method not only enriched AE signal processing theory, but it also provided an effective means for solving welding crack AE detection and ensuring structure quality. Research of the noise reduction method has certain significance in theoretical and practical engineering regarding AE signal processes and application fields.

## Figures and Tables

**Table 1:** Welding process parameters.

Experiment Number	Welding Speed (mm/s)	Peak Value Current (A)	Base Value Curren t(A)	Peak Voltage (V)	Base Value Voltage (V)	Duty Cycle (%)	Frequency (Hz)
1	6	100	50	24	14	50	6

**Table 2:** The parameters of acoustic emission (AE) system.

Parameter Term	Sampling Frequency /kHz	Sampling Length	Hit Dimension time /us	Parameter Threshold/db	Hit Locking time /us
Setting value	2500	2048	1000	45	2000
